# Supplementary material for: Modular Site-Specific Conjugation of Nanobodies Using Two Co-Associating Tags
Source: Int J Mol Sci. 2022 Nov 19;23(22):14405. doi: 10.3390/ijms232214405 (PMC9696751; doi:10.3390/ijms232214405)
Supplement: Supplementary file 1 [file ijms-23-14405-s001.zip › ijms-2014972-supplementary.pdf]

Supporting Information

Modular Site-Specific Conjugation of Nanobodies Using Two Co-Associating Tags

Eric Moeglin, Lina Barret, Bruno Chatton and Mariel Donzeau

mariel.donzeau@unistra.fr

Gene and protein sequences

Nano-HER2-E3

ATGGAAGTTCAACTGGTGGAATCGGGCGGTGGTCTGGTTCAAGCGGGCGGCTCACTGCGTCT
GTCCTGTGCTACCTCGGGCATCACGTTTATGCGTTATGCACTGGGTTGGTACCGTCAGAGCC
CGGGTAAACAACGTGAAATGGTTGCAAGTATTAACTCCGGCGGTACCACGAATTATGCTGAT
TCAGTCAAAGGCCGTTTTACCATCTCGCGCGACAACGCAAAAAATACGGTGTACCTGCAGAT
GAACAGTCTGAAACCGGAAGATACCGCGGTCTATTACTGCAATGCCCGCTGGGTGAAACCGC
AATTCATTGACAACAATTATTGGGGCCAGGGTACCCAAGTGACGGTTAGCTCTgcggccGCA
ACTAGTGAACAAAAACTCATCTCAGAAGAGGATCTGAATGctagCACGCCACTGGGTGACAC
GACTCATACCAGCGGTaacaacaccagctcctctccccagccaaagaagaaaccactggatg
gagaatatttcacccttcagatccgtgggcgtgagcgcttcgagatgttccgaGAGctgaat
GAGgccttggaactcGAGgatgcccaggctgggaaggagccagggGGTTCAGGCGGAGCTCC
ACATCACCATCATCACCATTAA

MEVQLVESGGGLVQAGGSLRLSCATSGITFMRYALGWYRQSPGKQREMVASINSGGTTNYAD
SVKGRFTISRDNAKNTVYLQMNSLKPEDTAVYYCNARWVKPQFIDNNYWGQGTQVTVSSAAA
TSEQKLISEEDLNASTPLGDTTHTSGNNTSSSPQPKKKPLDGEYFTLQIRGRERFEMFRELN
EALELEDAQAGKEPGGSGGAPHHHHHH

mScarlet-K3

ATGGCTAGTCACCATCACCATCACCATGCTGCCATGGTGAGCAAGGGCGAGGCAGTGATCAA
GGAGTTCATGCGGTTCAAGGTGCACATGGAGGGCTCCATGAACGGCCACGAGTTCGAGATCG
AGGGCGAGGGCGAGGGCCGCCCCTACGAGGGCACCCAGACCGCCAAGCTGAAGGTGACCAAG
GGTGGCCCCCTGCCCTTCTCCTGGGACATCCTGTCCCCTCAGTTCATGTACGGCTCCAGGGC
CTTCACCAAGCACCCCGCCGACATCCCCGACTACTATAAGCAGTCCTTCCCCGAGGGCTTCA
AGTGGGAGCGCGTGATGAACTTCGAGGACGGCGGCGCCGTGACCGTGACCCAGGACACCTCC
CTGGAGGACGGCACCCTGATCTACAAGGTGAAGCTCCGCGGCACCAACTTCCCTCCTGACGG
CCCCGTAATGCAGAAGAAGACAATGGGCTGGGAAGCGTCCACCGAGCGGTTGTACCCCGAGG
ACGGCGTGCTGAAGGGCGACATTAAGATGGCCCTGCGCCTGAAGGACGGCGGCCGCTACCTG
GCGGACTTCAAGACCACCTACAAGGCCAAGAAGCCCGTGCAGATGCCCGGCGCCTACAACGT
CGACCGCAAGTTGGACATCACCTCCCACAACGAGGACTACACCGTGGTGGAACAGTACGAAC
GCTCCGAGGGCCGCCACTCCACCGGCGGCATGGACGAGCTGTACAAGGCTAGCGGTAACAAC
ACCAGCTCCTCTCCCCAGCCAAAGAAGAAACCACTGGATGGAGAATATTTCACCCTTCAGAT
CCGTGGGCGTGAGCGCTTCGAGATGTTCCGAAAACTGAATAAGGCCTTGGAACTCAAGGATG
CCCAGGCTGGGAAGGAGCCAGGGGGTTAA

MASHHHHHHAAMVSKGEAVIKEFMRFKVHMEGSMNGHEFEIEGEGEGRPYEGTQTAKLKVTK
GGPLPFSWDILSPQFMYGSRAFTKHPADIPDYYKQSFPEGFKWERVMNFEDGGAVTVTQDTS
LEDGTLIYKVKLRGTNFPPDGPVMQKKTMGWEASTERLYPEDGVLKGDIKMALRLKDGGRYL
ADFKTTYKAKKPVQMPGAYNVDRKLDITSHNEDYTVVEQYERSEGRHSTGGMDELYKASGNN
TSSSPQPKKKPLDGEYFTLQIRGRERFEMFRKLNKALELKDAQAGKEPGG

eGFP-K3

ATGGCTAGTCACCATCACCATCACCATGCTAGGATGGTGAGCAAGGGCGAGGAGCTGTTCAC
CGGGGTGGTGCCCATCCTGGTCGAGCTGGACGGCGACGTAAACGGCCACAAGTTCAGCGTGT
CCGGCGAGGGCGAGGGCGATGCCACCTACGGCAAGCTGACCCTGAAGTTCATCTGCACCACC
GGCAAGCTGCCCGTGCCCTGGCCCACCCTCGTGACCACCCTGACCTACGGCGTGCAGTGCTT
CAGCCGCTACCCCGACCACATGAAGCAGCACGACTTCTTCAAGTCCGCCATGCCCGAAGGCT
ACGTCCAGGAGCGCACCATCTTCTTCAAGGACGACGGCAACTACAAGACCCGCGCCGAGGTG
AAGTTCGAGGGCGACACCCTGGTGAACCGCATCGAGCTGAAGGGCATCGACTTCAAGGAGGA
CGGCAACATCCTGGGGCACAAGCTGGAGTACAACTACAACAGCCACAACGTCTATATCATGG
CCGACAAGCAGAAGAACGGCATCAAGGTGAACTTCAAGATCCGCCACAACATCGAGGACGGC
AGCGTGCAGCTCGCCGACCACTACCAGCAGAACACCCCCATCGGCGACGGCCCCGTGCTGCT
GCCCGACAACCACTACCTGAGCACCCAGTCCGCCCTGAGCAAAGACCCCAACGAGAAGCGCG
ATCACATGGTCCTGCTGGAGTTCGTGACCGCCGCCGGGATCACTCTCGGCATGGACGAGCTG
TACGAAGCTAGCGGTAACAACACCAGCTCCTCTCCCCAGCCAAAGAAGAAACCACTGGATGG
AGAATATTTCACCCTTCAGATCCGTGGGCGTGAGCGCTTCGAGATGTTCCGAAAACTGAATA
AGGCCTTGGAACTCAAGGATGCCCAGGCTGGGAAGGAGCCAGGGGGTTAA

MASHHHHHHARMVSKGEELFTGVVPILVELDGDVNGHKFSVSGEGEGDATYGKLTLKFICTT
GKLPVPWPTLVTTLTYGVQCFSRYPDHMKQHDFFKSAMPEGYVQERTIFFKDDGNYKTRAEV
KFEGDTLVNRIELKGIDFKEDGNILGHKLEYNYNSHNVYIMADKQKNGIKVNFKIRHNIEDG
SVQLADHYQQNTPIGDGPVLLPDNHYLSTQSALSKDPNEKRDHMVLLEFVTAAGITLGMDEL
YEASGNNTSSSPQPKKKPLDGEYFTLQIRGRERFEMFRKLNKALELKDAQAGKEPGG

K3 synthetic peptide

CALNNGEYFTLQIRGRERFEMFRKLNKALELKDAQA

Table S1 : Biochemical-properties of the different constructs

	Number of aa	Monomer MW (kDa)	pI	Oligomerization state	MW of the complex (kDa)	Calculated MW by SEC
**Nano-HER2-E3**	213	23.50	6.88	Homodimer	47	70
**eGFP-K3**	305	35	6.80	Homotetramer	138	160
**mScarlet-K3**	302	34	8.60	Homodimer	136	155
**K3**	35	4	10.11	Homotetramer	16	-
**Nano-HER2-E3/eGFP-K3**	n.a	n.a	n.d	Heterotetramer	115	175
**Nano-HER2-E3/mScarlet-K3**	n.a	n.a	n.d	Heterotetramer	115	-
**Nano-HER2-E3/ K3**	n.a	n.a	n.d	Heterotetramer	76	70

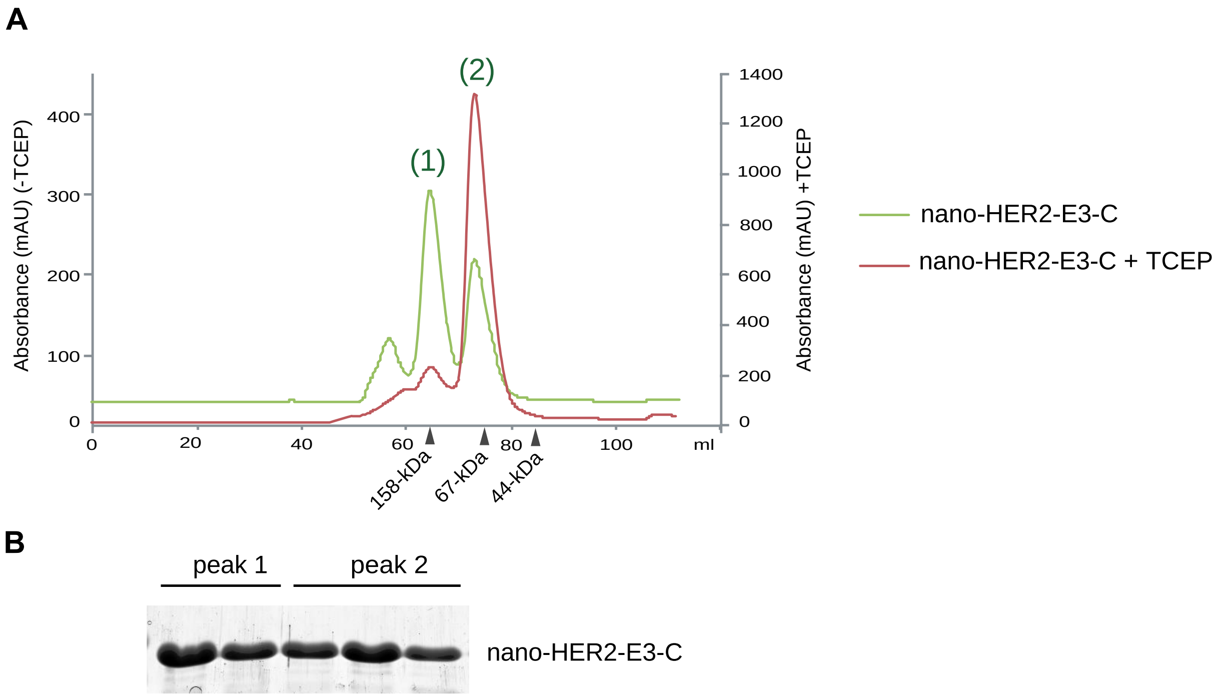

Figure S1: Purification of the nano-HER2-E3-C. (A) Purification of the nano-HER2-E3-C with (red) or without (green) TCEP on Hiload 16/160 Superdex 200pg after IMAC purification. (B) The two major peaks of nano-HER2-C without TCEP (1 and 2) were loaded on SDS-PAGE and stained by coomassie blue.

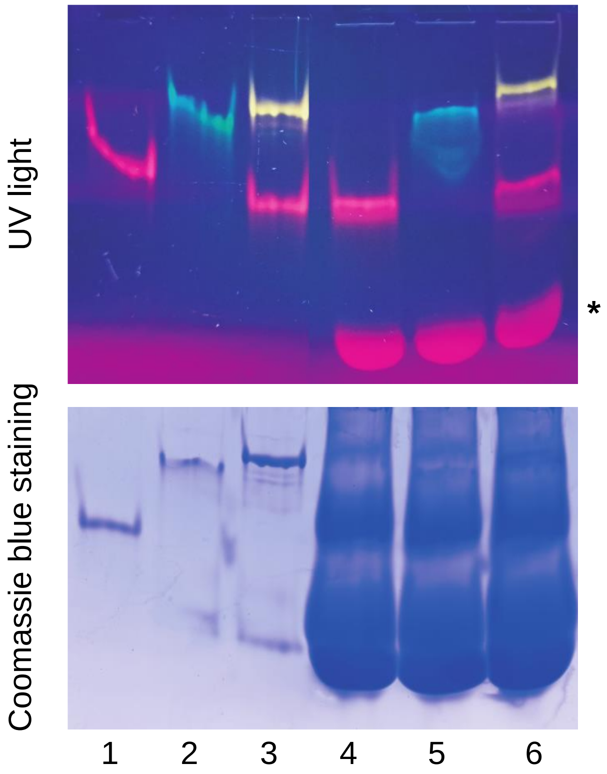

Figure S2: Complex formation in Serum. The eGFP-K3 purified protein was incubated with mCherry-E3 in PBS (lanes 1-3) or in the presence of 85 % foetal bovin serum (FBS) (lanes 4-6). The fluorescent moieties were revealed using UV light (upper panel) and FBS total proteins by Coomassie blue staining (lower panel): homotetramer mCherry-E3 (purple: lanes 1 and 4), eGFP-K3 (green: lanes 2 and 5), and heterotetramer mCherry-E3/eGFP-K3 (yellow: lanes 3 and 6). Intrinsic fluorescence of the Bovin Serum Albumin (BSA) is clearly visualized (upper panel, asterix).

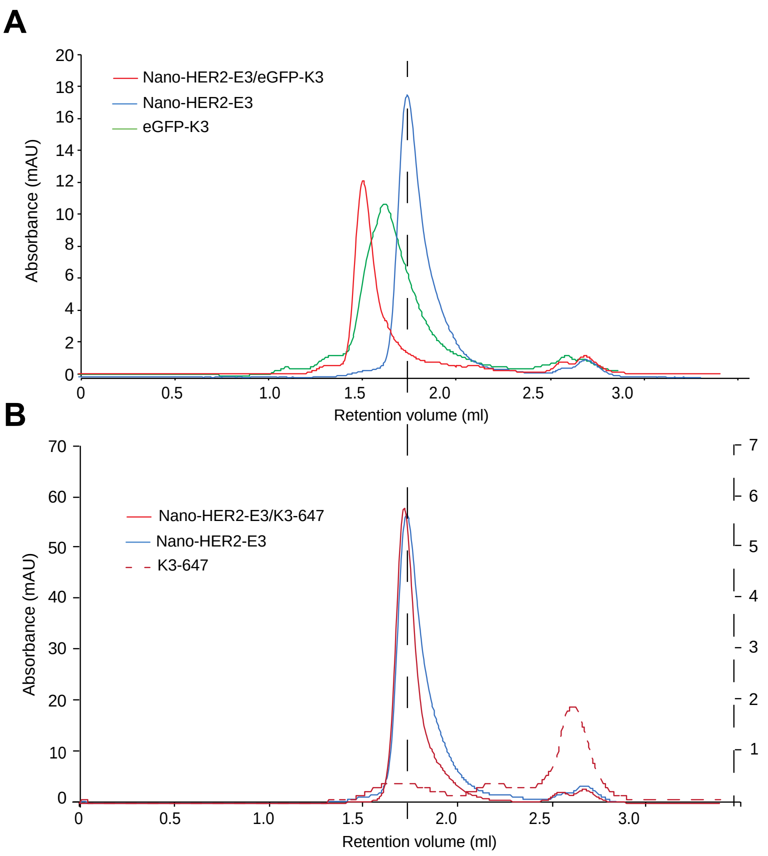

Figure S3: Analysis of complex formation. (A) Purified nano-HER2-E3 (blue), eGFP-K3 (green) and the heterotetrameric complex (red dashed line) or (B) nano-HER2-E3 (blue), mCherry-K3 (red) and the heterotetrameric complex (red dashed line) were mixed in equimolar amounts and analyzed by SEC.

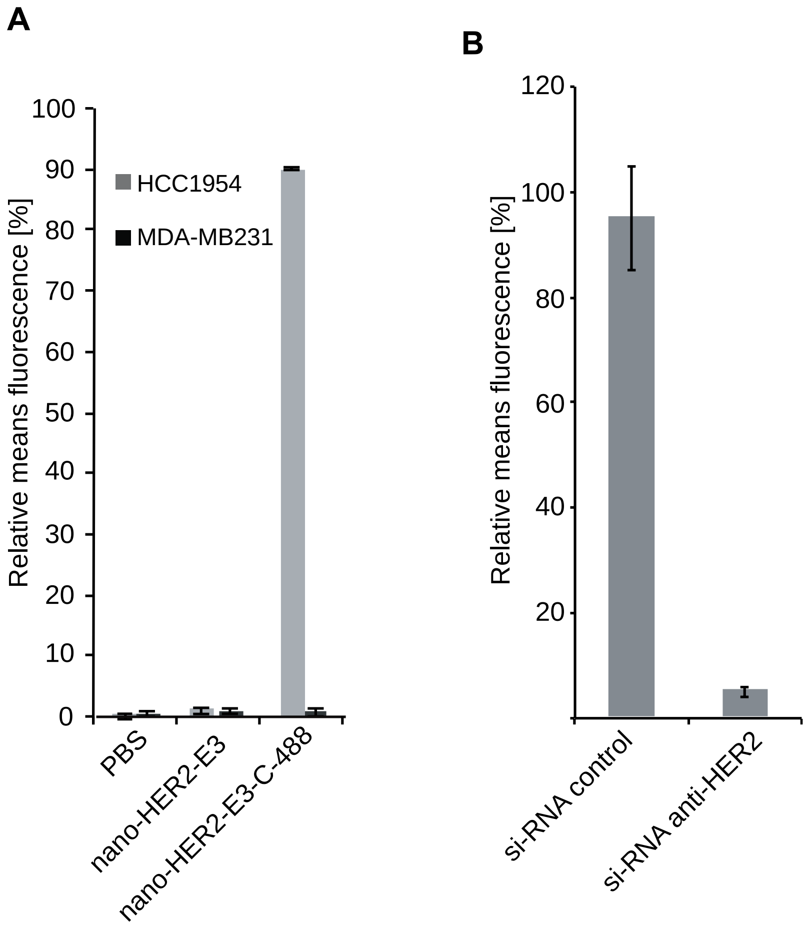

Figure S4: Analysis by flow cytometry of with the nano-HER2-E3-C-488 on HCC1954 and MDA-MB231 cell lines as indicated (A) or on HCC1954 treated with siRNA anti-HER2 or with a siRNA control (B). Relative means of fluorescence in % are represented.

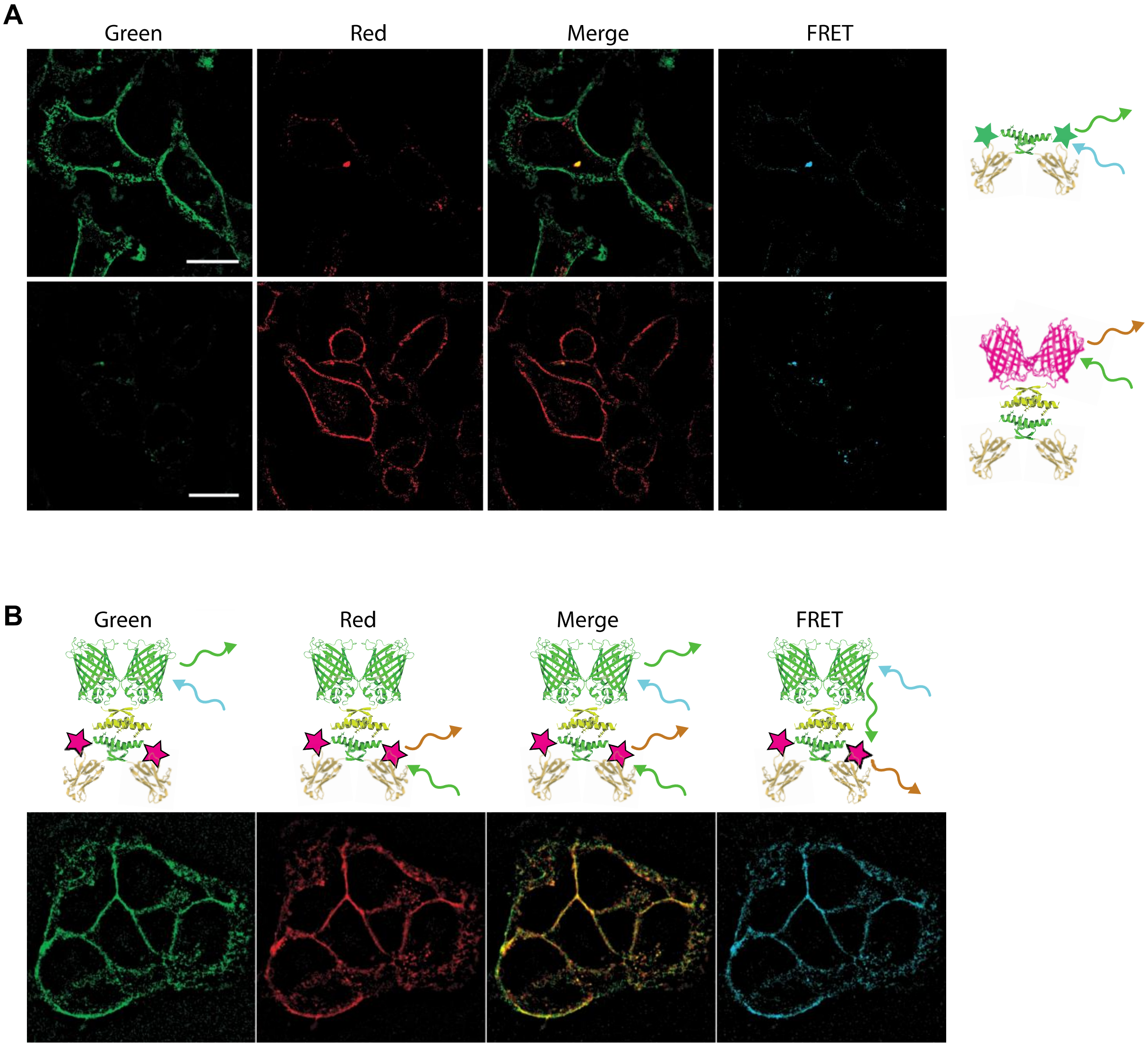

Figure S5: Complex formation at the cell surface receptor (A) control of the isolated fluorescent signals used in the FRET experiment. Upper panel: nano-HER2-E3-C-488, lower panel: nano-HER2-E3/mScarlet-K3. (B) FRET between and nano-HER2-E3-C-568 and eGFP-K3. HCC1954 cells were incubated with different complexes after fixation, as indicated. Images were taken by confocal microscopy and analyzed with Image J. Cartoons represent the complexes. Scale bar represent 10 µm.

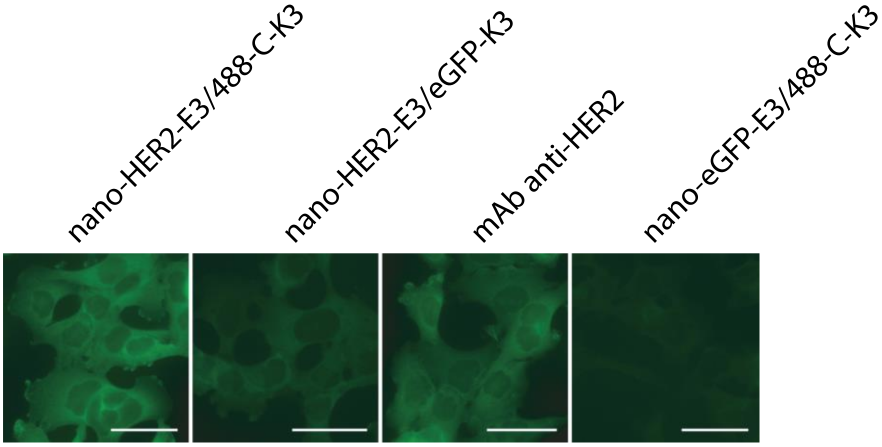

Figure S6: Indirect immmunofluorescence. Fixed HCC1954 cells were incubated with either different Alexa-Fluor-488 labeled complexes or with an anti-HER2 mAb followed by a secondary Alexa-488 secondary mAb, as indicated. Images were taken by confocal microscopy and analyzed with Image J. Cartoons represent the complexes. Scale bar represent 50 µm.
